# A kernel-based approach for detecting outliers of high-dimensional biological data

**DOI:** 10.1186/1471-2105-10-S4-S7

**Published:** 2009-04-29

**Authors:** Jung Hun Oh, Jean Gao

**Affiliations:** 1 Department of Computer Science and Engineering, The University of Texas, Arlington, Texas, USA

## Abstract

**Background:**

In many cases biomedical data sets contain outliers that make it difficult to achieve reliable knowledge discovery. Data analysis without removing outliers could lead to wrong results and provide misleading information.

**Results:**

We propose a new outlier detection method based on Kullback-Leibler (KL) divergence. The original concept of KL divergence was designed as a measure of distance between two distributions. Stemming from that, we extend it to biological sample outlier detection by forming sample sets composed of nearest neighbors. KL divergence is defined between two sample sets with and without the test sample. To handle the non-linearity of sample distribution, original data is mapped into a higher feature space. We address the singularity problem due to small sample size during KL divergence calculation. Kernel functions are applied to avoid direct use of mapping functions. The performance of the proposed method is demonstrated on a synthetic data set, two public microarray data sets, and a mass spectrometry data set for liver cancer study. Comparative studies with Mahalanobis distance based method and one-class support vector machine (SVM) are reported showing that the proposed method performs better in finding outliers.

**Conclusion:**

Our idea was derived from Markov blanket algorithm that is a feature selection method based on KL divergence. That is, while Markov blanket algorithm removes redundant and irrelevant features, our proposed method detects outliers. Compared to other algorithms, our proposed method shows better or comparable performance for small sample and high-dimensional biological data. This indicates that the proposed method can be used to detect outliers in biological data sets.

## Background

Outlier detection is an active research area that has many applications such as network intrusion detection [1], fraud detection [2] and biomedical data analysis [3]. In particular, outliers caused from instrument error or human error in the biomedical data analysis such as biomarker selection and disease diagnosis could deeply degrade the performance of the data analysis. Therefore, prior to the analysis, during preprocessing it is imperative to remove outliers to prevent wrong results. To detect such anomalous observations from normal ones, data mining techniques are widely used.

Outlier detection has been studied by researchers using a diversity of approaches. Statistical methods often view objects that are located relatively far from the center of the data distribution as outliers. Several distance measures were implemented. The Mahalanobis distance is the most commonly used multivariate outlier criterion. Based on Akaike's Information Criterion (AIC), Kadota *et al*. developed a method for detecting outliers, which is free from a significance level [4]. Knorr and Ng introduced a distance-based approach in which outliers are those objects for which there are less than *k *points within a given threshold in the input data set [5,6]. Angiulli *et al*. proposed a distance-based outlier detection method which finds the top outliers and provides a subset of the data set, called outlier detection solving set, that can be used to predict if new unseen objects are outliers [7]. Distance-based strategies are advantageous since model learning is not required. As an alternative, clustering algorithms can be used for outlier detection in which objects that do not belong to any cluster are regarded as outliers. Wang and Chiang proposed an effective cluster validity measure with outlier detection and cluster merging strategies for support vector clustering (SVC) [8]. The validity measure is capable of finding suitable values for the kernel parameter and soft margin constant. Based on these parameters, SVC algorithm can identify the ideal cluster number and increase robustness to outliers and noises. Schölkopf proposed a method of adapting support vector machine (SVM) to one-class classification problems [9]. Manevitz and Yousef presented two versions using the one-class SVM, both of which can identify outliers: Schölkopf's method and their proposed suggestion [10]. In such methods, after mapping the original samples into a feature space using an appropriate kernel function, the origin is referred to as the second class. In the feature space, samples close to the origin or lying on the standard subspace such as axes are regarded as outliers. Bandyopadhyay and Santra applied a genetic algorithm to the outlier detection problem in a lower dimensional space of a given data set, dividing these spaces into grids and efficiently computing the sparsity factor of the grid [11]. Aggarwal and Yu studied the problem of outlier detection for high-dimensional data, which works by finding lower dimensional projections [12]. Malossini *et al*. proposed two methods for detecting potential labeling errors: Classification-stability algorithm (CL-stability) and Leave-One-Out-Error-sensitivity algorithm (LOOE-sensitivity) [13]. In CL-stability, the stability of the classification of a sample is evaluated with a small perturbation of the other samples. LOOE-sensitivity was derived from the fact that if a sample is mislabeled, flipping the label of the sample should improve the prediction power.

In this paper, we propose a new outlier detection method based on KL divergence [14]. Due to the possible non-linearity of data structure, we deal with this problem in a higher feature space rather than the original space. Several issues arise after data mapping such as singularity because of small sample size versus high feature dimension. We address the computational issues and show the effectiveness of the proposed approach, KL divergence for outlier detection (KLOD).

## Methods

### Markov blanket

Markov blanket algorithm proposed by Koller and Sahami is a cross-entropy based technique to identify redundant and irrelevant features [15]. Let **F **be a full set of features and **M **⊆ **F **be a subset of features which does not contain feature *F*_*i*_. Then, **M **is called a Markov blanket for *F*_*i *_if *F*_*i *_is conditionally independent of **F **- **M**-{*F*_*i*_} given **M**. Generally, the Markov blanket **M**_*i *_of *F*_*i *_is defined as a subset of features that consists of some features that have the highest Pearson correlation with *F*_*i*_. To evaluate the closeness between *F*_*i *_and its Markov blanket **M**_*i*_, the following expected cross-entropy Δ is estimated:

(1) $$\Delta (F_{i}|M_{i})=\sum_{f_{M_{i}},f_{i}}P(M_{i}=f_{M_{i}},F_{i}=f_{i})\times D(P(c|M_{i}=f_{M_{i}},F_{i}=f_{i})||P(c|M_{i}=f_{M_{i}})),$$

where **f**_**M***i *_and *f*_*i *_are feature values to **M**_*i *_and *F*_*i*_, respectively, *c *is the class label, and *D*(.||.) represents the cross-entropy (a.k.a. Kullback-Leibler divergence). For each feature, Δ value is computed and a feature with the smallest Δ value is eliminated from the whole feature set. With the remaining features, the procedure is repeated until a predefined number of features remains.

### Kullback-Leibler (KL) divergence

KL divergence, widely used in information theory, is adopted in Markov blanket as a core component. As shown in Markov blanket, KL divergence represents a measure of the distance between two probability distributions [16], i.e., for two probability densities *p*(**x**) and *q*(**x**), the KL-divergence is defined as

(2) $$D_{\text{KL}}(p||q)=\int_{x}p(x)\log\frac{p(x)}{q(x)}dx.$$

Suppose that N(*μ*, Σ) is a multivariate Gaussian distribution defined as

(3) $$N(\mu ,\sum )=\frac{1}{\sqrt{{\left(2\pi \right)}^{m}|\sum |}}\exp\left(-\frac{1}{2}{\left(x-\mu \right)}^{\text{T}}\sum^{-1}(x-\mu )\right),$$

where **x **∈ $R^{m}$ and |Σ| is the determinant of covariance matrix Σ. Given two different probability density functions, *p*(**x**) = N_1_(*μ*_1_, Σ_1_) and *q*(**x**) = N_2_(*μ*_2_, Σ_2_), the KL divergence is defined as

(4) $$D_{KL}(N_{1}||N_{2})=\frac{1}{2}\{{\left(\mu_{1}-\mu_{2}\right)}^{T}\sum_{2}^{-1}(\mu_{1}-\mu_{2})+log\frac{\left|\sum_{2}\right|}{\left|\sum_{1}\right|}+tr[\sum_{1}\sum_{2}^{-1}-I_{m}]\}.$$

### Concept of KL divergence for outlier detection (KLOD)

In Markov blanket, based on KL divergence, after calculating Δ value of Eq. (1) for each feature, a feature with the lowest Δ value is considered to be the most redundant. Using KL divergence, our new outlier detection method, called KLOD, employs similar strategy to the Markov blanket, i.e., while Markov blanket algorithm detects redundant and irrelevant features, our method identifies outliers. In KLOD, each sample **x**_*i *_has a sample set that consists of *t *samples close to the **x**_*i*_. To calculate the distance between samples, Euclidean metric is used. More specifically, we define two sample sets, i.e., **S**_1 _and **S**_2_: **S**_2 _is a sample set close to **x**_*i *_in Euclidean distance and the other set **S**_1 _consists of **x**_*i *_and all samples in **S**_2_. The similarity, $D_{x_{i}}$(**S**_1_||**S**_2_), between **S**_1 _and **S**_2 _for each sample can be measured by using KL divergence, where 1 ≤ *i *≤ *n *and *n *is the total number of samples in the data set. Intuitively, in our strategy, a sample **x**_*i *_with the largest *D *is regarded as an outlier.

(5) $$o=\arg\max_{1\leq i\leq n}D_{x_{i}}$$

Given a data set with nonlinear data structure, if we model the linearity for the data set, it will cause our strategy to fail. Here, we focus on modeling the nonlinearity. Accordingly, with a mapping function *ϕ*, the original space is mapped into a higher dimensional feature space. Let $S_{1}^{\Phi }$ and $S_{2}^{\Phi }$ denote the two sample sets in the feature space in which we compute the similarity *D*($S_{1}^{\Phi }$||$S_{2}^{\Phi }$) between $S_{1}^{\Phi }$ and $S_{2}^{\Phi }$. For each sample, its *D*($S_{1}^{\Phi }$||$S_{2}^{\Phi }$) is calculated. A sample which has the largest *D*($S_{1}^{\Phi }$||$S_{2}^{\Phi }$) is referred to as an outlier.

**Figure 1:**
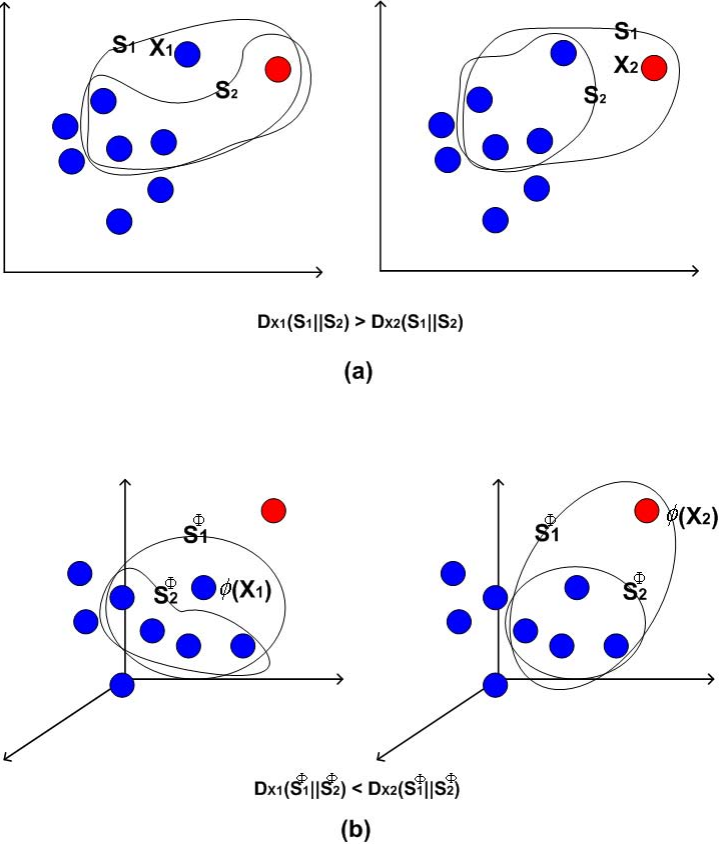
**Outlier detection in a high feature space**. Suppose that the red dot is a real outlier which is the farthest one from the majority of data. (a) in the original space, **x**_1 _is regarded as an outlier. (b) in the higher feature space, **x**_2 _is correctly detected as an outlier.

Please see an example in Figure 1. However, the calculation leads to several important issues to be considered, such as kernel trick, singularity problem, and calculation of KL divergence in the feature space. In the following sections, we will describe them.

### Kernel function

Suppose that {**x**_1_, **x**_2_, ⋯ **x**_*n*_} are the given samples in the original space. After mapping the samples into a higher feature space by a nonlinear mapping function *ϕ*, the samples in the feature space are observed as Φ_*mxn *_= [*ϕ*(**x**_1_), *ϕ *(**x**_2_), ⋯, *ϕ *(**x**_*n*_)] where *m *is the number of features. Denote **K **as follows:

(6) **K **= Φ^**T**^Φ.

The calculation can be performed using kernel trick, i.e., the *ij*th element, *ϕ *(**x**_*i*_)^**T**^*ϕ *(**x**_*j*_), of the **K **matrix can be computed as a kernel function *k*(**x**_*i*_, **x**_*j*_). In literatures, the polynomial kernel and the Gaussian kernel are the most widely used kernel functions. In this study, the Gaussian kernel function is used:

(7) $$k(x,y)=\exp\left(-\frac{||x-y|{|}^{2}}{2\sigma^{2}}\right),$$

where *σ *controls the kernel width. Similar to Eq. (6), we define **K**_*ij *_as follows:

(8) $$K_{ij}=\Phi_{i}^{T}\Phi_{j},$$

where if *i *≠ *j*, Φ_*i *_and Φ_*j *_are different sample sets in the feature space; if *i *= *j*, **K**_*ij *_is equivalent to the definition of **K**. Indeed, the feature space and the mapping function may not be explicitly known. However, once the kernel function is known, we can easily deal with the nonlinear mapping problem by replacing the mapping functions by the kernel functions.

KL divergence equation is composed of mean and covariance components. The mean and the covariance matrix in the feature space are estimated as

(9) $$\hat{\mu }=\frac{1}{n}\sum_{i=1}^{n}\phi (x_{i})=\Phi s,$$

(10) $$\hat{\Sigma }=\frac{1}{n}\sum_{i=1}^{n}(\phi (x_{i})-\mu ){\left(\phi (x_{i})-\mu \right)}^{T}=\Phi JJ^{T}\Phi^{T},$$

where $s_{n\times 1}=\frac{1}{n}{\vec{1}}^{T},J=\frac{1}{\sqrt{n}}(I_{n}-s\vec{1})$ and $\vec{1}$ = [1, 1, ⋯, 1]. Then, an *m *× *n *matrix **W **is denoted as

(11) $$W=\Phi J=\frac{1}{\sqrt{n}}[(\phi (x_{1})-\mu ),\cdots ,(\phi (x_{n})-\mu )].$$

### Singularity problem

The covariance matrix in Eq. (10) is rank-deficient due to the small number of samples against the number of features. This problem, called singularity problem, makes it impossible to calculate the inverse of the covariance matrix. To overcome the problem, several methods have been proposed. In this study, we make use of a simple regularized approximation in which some positive constant values are added to the diagonal elements of the covariance matrix [17]. Therefore, the modified covariance matrix is of full rank, hence nonsingular. Let **C **denote

(12) $$\begin{array}{c} C=\Phi JJ^{T}\Phi^{T}+\rho I_{m},\\ =WW^{T}+\rho I_{m},\\ =\Phi R\Phi^{T}+\rho I_{m}, \end{array}$$

where **R **= **JJ**^**T**^, *ρ *> 0, and **I**_*m *_is an identity matrix. In this study, *ρ *= 1 is used. Then, the inversion of the matrix **C **can be computed by using *Woodbury formula*:

(13) $$\begin{array}{c} C^{-1}={\left(\rho I_{m}+\Phi JJ^{T}\Phi^{T}\right)}^{-1},\\ ={\left(\rho I_{m}+WW^{T}\right)}^{-1},\\ =\rho^{-1}(I_{m}-\rho^{-1}W{\left(I_{n}+\rho^{-1}W^{T}W\right)}^{-1}W^{T}),\\ =\rho^{-1}(I_{m}-W{\left(\rho I_{n}+W^{T}W\right)}^{-1}W^{T}),\\ =\rho^{-1}(I_{m}-\Phi JM^{-1}J^{T}\Phi^{T}),\\ =\rho^{-1}(I_{m}-\Phi B\Phi^{T}), \end{array}$$

where **B **= **JM**^-1^**J**^T ^and **M **= *ρ***I**_*n *_+ **W**^**T**^**W **= *ρ ***I**_*n *_+ **J**^**T**^Φ^**T**^Φ**J **= *ρ***I**_*n *_+ **J**^**T**^**KJ**.

**Definition **(Woodbury formula): Let **A **be a square *r *× *r *invertible matrix, where **U **and **V **are two *r *× *k *matrices with *k *≤ *r*. Assume that a *k *× *k *matrix Σ = **I**_*k *_+ *β ***V**^**T**^**A**^-1^**U**, in which **I**_*k *_denotes a *k *× *k *identity matrix and *β *is an arbitrary scalar, is invertible. Then

(**A **+ *β ***UV**^**T**^)^-1 ^= **A**^-1 ^- *β ***A**^-1^**UΣ**^-1^**V**^**T**^**A**^-1^.

### Calculation of KL divergence

Suppose that $S_{1}^{\Phi }$ and $S_{2}^{\Phi }$ are two sample sets in the feature space as mentioned in section. We know that the covariance matrices for both sets are singular. Let **C**_1 _and **C**_2 _denote the approximated covariance matrices for $S_{1}^{\Phi }$ and $S_{2}^{\Phi }$, respectively, where the size of $S_{1}^{\Phi }$ is one larger than that of $S_{2}^{\Phi }$. Also, let *μ*_1 _and *μ*_2 _be mean matrices for $S_{1}^{\Phi }$ and $S_{2}^{\Phi }$, respectively. Therefore, KL divergence for $S_{1}^{\Phi }$ and $S_{2}^{\Phi }$ is expressed as follows:

(14) $$2D_{KL}(N_{1}||N_{2})={\left(\mu_{1}-\mu_{2}\right)}^{T}C_{2}^{-1}(\mu_{1}-\mu_{2})+log\frac{\left|C_{2}\right|}{\left|C_{1}\right|}+tr|C_{1}C_{2}^{-1}]-m.$$

The KL divergence above is composed of three terms, i.e.,

$$\{\begin{array}{l} {\left(\mu_{1}-\mu_{2}\right)}^{T}C_{2}^{-1}(\mu_{1}-\mu_{2})\\ log\frac{\left|C_{2}\right|}{\left|C_{1}\right|}\\ tr[C_{1}C_{2}^{-1}]. \end{array}$$

It should be noted that as shown in Eq. (9), Eq. (12) and Eq. (13), *μ*_*i*_, **C**_*i *_and $C_{i}^{-1}$ (*i *= 1 or 2) have mapping functions rather than kernel functions.

Here, we will show how each term can be expressed by kernel functions instead of mapping functions. The first term consists of four sub-terms,

$${\left(\mu_{1}-\mu_{2}\right)}^{T}C_{2}^{-1}(\mu_{1}-\mu_{2})=\mu_{1}^{T}C_{2}^{-1}\mu_{1}+\mu_{2}^{T}C_{2}^{-1}\mu_{2}-\mu_{1}^{T}C_{2}^{-1}\mu_{2}-\mu_{2}^{T}C_{2}^{-1}\mu_{1}.$$

Substituting Eq. (9) and Eq. (13) into each sub-term $\mu_{i}^{T}C_{j}^{-1}\mu_{k}$, we have

(15) $$\begin{array}{c} \mu_{i}^{T}C_{j}^{-1}\mu_{k}=s_{i}^{T}\Phi_{i}^{T}\rho^{-1}(I_{m}-\Phi_{j}B_{j}\Phi_{j}^{T})\Phi_{k}s_{k},\\ =\rho^{-1}(s_{i}^{T}K_{ik}s_{k}-s_{i}^{T}K_{ij}B_{j}K_{jk}s_{k}),\\ =\rho^{-1}\theta_{ijk}. \end{array}$$

As a result of the effort, all mapping functions in the first term are replaced with kernel functions. Before dealing with the second term, we want to introduce the following three properties of determinant that are essential in the calculation of the second term.

### Properties of determinant

(a) If **A **is an *r*-by-*r *matrix, **det**|*d***A**| = **det**|*d***I**_*r*_**A**| = *d*^*r*^**det**|**A**|.

(b) If **A **and **B **are *k*-by-*r *matrices, **det**|**I**_*k *_+ **AB**^**T**^| = **det**|**I**_*r *_+ **B**^**T**^**A**|.

(c) If **A **is invertible, **det**|**A**^-1^| = **1/det**|**A**|.

In the second term, we should compute the determinant of **C**(**C**_1 _or **C**_2_). Instead of directly calculating the determinant of **C**, we try to obtain it through the determinant of **C**^-1^. That is,

(16) $$\begin{array}{llll} \left|C^{-1}\right| & = & |\rho^{-1}(I_{m}-\Phi B\Phi^{T})|, & \\ & = & \rho^{-m}|I_{m}-\Phi B\Phi^{T}|, & \text{by property }(\text{a})\\ & = & \rho^{-m}|I_{m}-Q\Phi^{T}|, & \\ & = & \rho^{-m}|I_{n}-\Phi^{T}Q|, & \text{by property }(\text{b})\\ & = & \rho^{-m}|I_{n}-\Phi^{T}\Phi B|, & \\ & = & \rho^{-m}|I_{n}-KB|, & \end{array}$$

where **Q **= Φ**B**. Here, by property (c), we can easily calculate |**C**|, i.e.,

(17) $$|C|=\frac{1}{\left|C^{-1}\right|}=\frac{\rho^{m}}{\left|I_{n}-KB\right|}.$$

By taking logarithm of |**C**|, we have

(18) $$log|C|=log\frac{\rho^{m}}{\left|I_{n}-KB\right|}=mlog\rho -log|I_{n}-KB|.$$

Note that the size of $S_{1}^{\Phi }$ is one larger than that of $S_{2}^{\Phi }$. If the size of $S_{2}^{\Phi }$ is *k*, the size of $S_{1}^{\Phi }$ becomes *k *+ 1.

Now we have the second term composed of kernel functions:

(19) $$\begin{array}{c} log\frac{\left|C_{2}\right|}{\left|C_{1}\right|}=log|C_{2}|-log|C_{1}|,\\ =log|I_{k+1}-K_{11}B_{1}|-log|I_{k}-K_{22}B_{2}|. \end{array}$$

The third term can be replaced with kernel functions using properties of trace:

(20) $$\begin{array}{lll} tr[C_{1}C_{2}^{-1}] & = & tr[(\Phi_{1}R_{1}\Phi_{1}^{T}+\rho I_{m})\rho^{-1}(I_{m}-\Phi_{2}B_{2}\Phi_{2}^{T})],\\ & = & \rho^{-1}tr[\Phi_{1}R_{1}\Phi_{1}^{T}]-\rho^{-1}tr[\Phi_{1}R_{1}\Phi_{1}^{T}\Phi_{2}B_{2}\Phi_{2}^{T}]+m-tr[\Phi_{2}B_{2}\Phi_{2}^{T}],\\ & = & \rho^{-1}tr[R_{1}K_{11}]-\rho^{-1}tr[R_{1}K_{12}B_{2}K_{21}]+m-tr[B_{2}K_{22}]. \end{array}$$

Successfully, we substitute all mapping functions in the three terms of KL divergence by kernel functions so that we can calculate KL divergence between two sample sets in the feature space.

## Results and discussion

To evaluate the performance of KLOD method, we performed several experiments using a synthetic data, two gene expression data sets, and a high-resolution mass spectrometry data. To obtain unbiased results, all experiments were repeated 30 times with 10-fold cross validation (CV) and the performance was averaged. The performance of KLOD was compared with one-class SVM and Mahalanobis distance based outlier detection methods. Given *n *samples, the Mahalanobis distance for each multivariate sample **x**_*i *_is as follows:

(21) $$D_{i}=\sqrt{{\left(x_{i}-\mu \right)}^{T}\Sigma^{-1}(x_{i}-\mu )}$$

where Σ and *μ *are the sample covariance matrix and sample mean vector, respectively. Samples with a large Mahalanobis distance are regarded as outliers.

### Results on synthetic data

**Figure 2:**
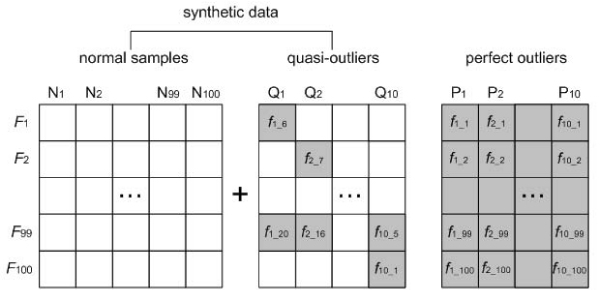
**Generation of a synthetic data**. This example shows a way used in this study to generate a synthetic data.

First, using a synthetic data, we evaluated KLOD to see the ability in detecting outliers. The synthetic data consists of 100 samples, denoted as **N**, each of which has 100 features generated from a mixture of Gaussian N (0, **I**). In addition, two sample sets called quasi-outlier set **Q **and perfect outlier set **P **were produced, each of which has 10 samples with 100 features, which were generated from a mixture of Gaussian N (0, **I**) and N (2, **I**), respectively. It is noted that **Q **was created from the same distribution as **N**. Here, we corrupted **Q **by changing the values in some features. To do so, some features from each sample in **P **were randomly selected. The values of the selected features replaced those of features randomly selected from the corresponding sample in **Q**. Finally, we merged **N **and **Q**, which were used as a synthetic data. Figure 2 illustrates an example of generating the synthetic data. In this experiment, we tested KLOD changing the number of corrupted features from 10 to 30 increasing by 2 and the size of a set, denoted as *t*, that consists of close samples of each sample from 5 to 20 increasing by 5. With the synthetic data, we measured how accurate our method is in identifying outliers in a way that the number of real outliers is counted out of the first 10 samples detected by KLOD.

**Figure 3:**
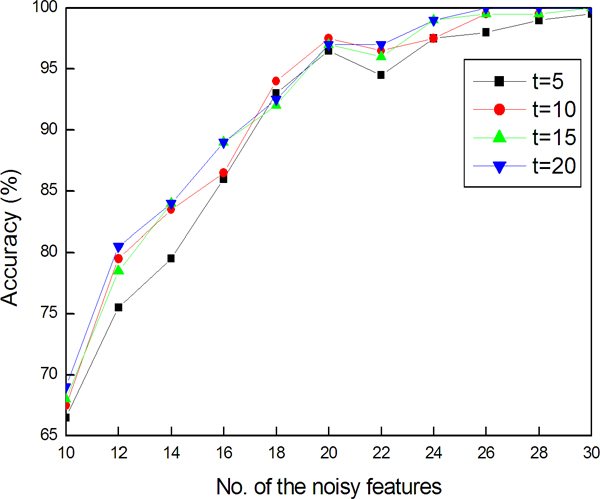
**Accuracy of detecting outliers on a synthetic data**. The data consists of 100 normal samples and 10 outliers, each having 100 features.

Figure 3 shows the experimental results. When the number of noisy features increases, the accuracy shows a tendency to increase as well. It should be noted that for all set sizes, when the number of noisy features is 18, an accuracy of over 90% was obtained. Particularly, for *t *= 10, 15 and 20, when the number of noisy features is 30, an accuracy of 100% was achieved.

### Performance evaluation after outlier removal

Before introducing the outlier removal for real biomedical data, we first introduce the performance evaluation metric we will use which is PCA (principal component analysis) + LDA (linear discriminant analysis). LDA maps the data into a very low dimensionality of *c *-1, where *c *is the number of classes. In the reduced space, a simple matching procedure is used for classification. However, in order to guarantee a non-degenerate result from LDA, before the LDA task, the dimensionality of the data must be reduced to at most *n *- *c *where *n *is the number of samples. Principal component analysis (PCA) is often used in the analysis of high dimensional data set. PCA performs a transformation of the original space into a lower dimensional space with little or no information loss while maximally preserving variance.

Lilien *et al*. used the PCA+LDA method in the analysis of mass spectrometry data sets [18]. In this framework, the PCA dimensionality-reduced samples are projected by LDA onto a hyperplane in the way of maximizing the between-class variance and minimizing the within-class variance of the projected samples. To evaluate the performance after outlier removal in our experiments, we employed the PCA+LDA strategy.

### Results on gene expression data sets

In this study, two public microarray data sets were used.

• The leukemia data set covers two types of acute leukemia: 47 acute lymphoblastic leukemia (ALL) samples and 25 acute myeloid leukemia (AML) samples with 7,129 genes. The data set is publicly available at [19].

• The colon data set contains 40 tumor and 22 normal colon tissues with 2,000 genes. The data set is available at [20].

In experiments with the two microarray data sets, specificity, sensitivity, and accuracy were measured using PCA+LDA classification strategy after removing outliers detected by KLOD with *t *= 10, Mahalanobis distance based method, and one-class SVM. We define the specificity as the ratio of correctly classified negatives to the actual number of negatives. For leukemia and colon microarray data sets, negatives are ALL and normal samples, respectively. For KLOD and Mahalanobis distance based method, the performance was measured after removing a sample having the largest distance from each class at each iteration. If the prediction rate (specificity or sensitivity) decreases more than a threshold *γ *compared to the prediction rate before the outlier removal, we stop the outlier detection in the corresponding class. In this study, we used *γ *= 0.5%. In contrast, for one-class SVM, after excluding all samples regarded as outliers in each class, the performance was assessed.

Table 1 shows the experimental results obtained using leukemia and colon microarray data sets. For the leukemia data set, KLOD achieved the best accuracy with 9 outliers (2 ALL and 7 AML samples).

**Table 1:** Performance after outlier detection in leukemia and colon data sets.

Data set	Measurements	Without outlier removal	After outlier removal		
			
			KLOD	Mahalanobis	One-class SVM
Leukemia	Specificity (%)	96.17	99.00	97.37	100
	Sensitivity (%)	95.60	99.44	100	95.24
	Accuracy (%)	95.97	99.13	98.28	98.33

	No. of the outliers	ALL	2	9	8
		AML	7	5	4

Colon	Specificity (%)	82.50	85.95	83.25	85.26
	Sensitivity (%)	88.25	94.43	85.90	94.17
	Accuracy (%)	86.21	91.25	85.00	91.09

	No. of the outliers	normal	1	2	3
		tumor	5	1	4

Mahalanobis distance based method and one-class SVM found 14 and 12 outliers, respectively. For the colon data set, KLOD found 6 outliers (1 normal and 5 tumor samples) with 84.95% specificity, 94.43% sensitivity, and 91.25% accuracy. It should be noted that the performance of Mahalanobis distance based method was degraded in terms of sensitivity and accuracy compared to the performance obtained using all samples without outlier removal, suggesting that outliers detected by Mahalanobis distance based method are unlikely to be real ones.

### Results on mass spectrometry data

To evaluate the effectiveness of KLOD, we also used a public mass spectrometry data for liver cancer study that consists of 201 spectra containing hepatocellular carcinoma (HCC) (78), cirrhosis (51), and health (72) [3]. From , we downloaded the binned spectra that have 23,846 peaks for each spectrum. To test outlier detection methods, only cirrhosis and HCC spectra were used as in [3]. By using t-test with the significance level of 0.05 in cirrhosis and HCC spectra, we selected 10,682 peaks. That is, the top 10,682 peaks selected by t-test with cirrhosis and HCC spectra were used in outlier detection methods. The same way as performed with the microarray data sets was employed. Here cirrhosis samples are regarded as negatives. As shown in Table 2, KLOD obtained slightly higher performance with the smallest number of outliers than Mahalanobis distance based method and one-class SVM. From the results in experiments using mass spectrometry and microarray data sets, it seems that one-class SVM detects more outliers than KLOD and Mahalanobis distance based method.

**Table 2:** Performance after outlier detection in liver cancer mass spectrometry data.

Measurements	Without outlier removal	After outlier removal		
		
		KLOD	Mahalanobis	One-class SVM
Specificity (%)	93.63	94.69	94.29	94.35
Sensitivity (%)	92.82	93.95	93.51	93.89
Accuracy (%)	93.14	94.23	93.82	94.07

No. of the outliers	Cirrhosis	3	2	5
	HCC	2	4	6

## Conclusion

We proposed a new outlier detection method based on KL divergence called KLOD. Our idea was derived from Markov blanket algorithm where redundant and irrelevant features are removed based on KL divergence. We tackled the outlier detection problem in a higher feature space after mapping the original data. The mapping leads to several issues. In particular, we showed how to calculate KL divergence in the higher feature space by using the properties of determinant and trace of matrix. To asses the usefulness of KLOD, we used a synthetic data and real life data sets. Compared to Mahalanobis distance based method and one-class SVM, KLOD achieved higher or comparable performance.

## Competing interests

The authors declare that they have no competing interests.

## Authors' contributions

JHO performed data analysis and wrote the manuscript. JG supervised the project and edited the paper.
